# Myeloproliferative neoplasms (MPNs) have a significant impact on patients’ overall health and productivity: the MPN Landmark survey

**DOI:** 10.1186/s12885-016-2208-2

**Published:** 2016-02-27

**Authors:** Ruben Mesa, Carole B. Miller, Maureen Thyne, James Mangan, Sara Goldberger, Salman Fazal, Xiaomei Ma, Wendy Wilson, Dilan C. Paranagama, David G. Dubinski, John Boyle, John O. Mascarenhas

**Affiliations:** St. Agnes Hospital, 900 S Caton Ave, Baltimore, MD 21229 USA; Weill Cornell Medical College, 525 E 68Th St Starr 341, New York, NY 10065 USA; Cancer Support Community, 165 W46th Street Suite 1002, New York, NY 10036 USA; Allegheny Health Network, 4815 Liberty, Mellon Suite 340, Pittsburgh, PA 15224 USA; Fred Hutchinson Cancer Research Center, 1100 Fairview Ave. North, LF-210, Seattle, WA 98109 USA; Incyte Corporation, 1801 Augustine Cut-Off, Wilmington, DE 19803 USA; Division of Hematology & Medical Oncology, Mayo Clinic Cancer Center, 13400 E. Shea Blvd, Scottsdale, AZ 85259 USA; University of Pennsylvania, Abramson Cancer Center, Perelman Center for Advanced Medicine, West Pavilion, 2nd Floor, 3400 Civic Center Boulevard, Philadelphia, PA 19104 USA; Yale School of Public Health, Laboratory of Epidemiology and Public Health, 60 College Street, Suite 406, New Haven, CT 06510 USA; ICF International, 530 Gaither Road, Suite 500, Rockville, MD 20850 USA; Icahn School of Medicine at Mount Sinai, Ruttenberg Treatment Center, 1470 Madison Avenue, 3rd Floor, New York, NY 10029 USA

**Keywords:** Polycythemia vera, Signs and symptoms, Quality of life, Activities of daily living

## Abstract

**Background:**

The Philadelphia chromosome−negative myeloproliferative neoplasms (MPN) myelofibrosis (MF), polycythemia vera (PV), and essential thrombocythemia (ET) negatively affect patient quality of life (QoL) and are associated with increased risk of mortality.

**Methods:**

The MPN Landmark survey was conducted from May to July 2014 in patients with MF, PV, or ET under active management in the United States. The survey assessed respondent perceptions of disease burden and treatment management and included questions on overall disease burden, QoL, activities of daily living, and work productivity. Outcomes were further analyzed by calculated (ie, not respondent-reported) prognostic risk score and symptom severity quartile.

**Results:**

The survey was completed by 813 respondents (MF, *n* = 207; PV, *n* = 380; ET, *n* = 226). The median respondent age in each of the 3 MPN subtypes ranged from 62 to 66 years; median disease duration was 4 to 7 years. Many respondents reported that they had experienced MPN-related symptoms ≥1 year before diagnosis (MF, 49 %; PV, 61 %; ET, 58 %). Respondents also reported that MPN-related symptoms reduced their QoL, including respondents with low prognostic risk scores (MF, 67 %; PV, 62 %; ET, 57 %) and low symptom severity (MF, 51 %; PV, 33 %; ET, 15 %). Many respondents, including those with a low prognostic risk score, reported that their MPN had caused them to cancel planned activities or call in sick to work at least once in the preceding 30 days (cancel planned activities: MF, 56 %; PV, 35 %; ET, 35 %; call in sick: MF, 40 %; PV, 21 %; ET, 23 %).

**Conclusions:**

These findings of the MPN Landmark survey support previous research about the symptom burden experienced by patients with MPNs and are the first to detail the challenges that patients with MPNs experience related to reductions in activities of daily living and work productivity.

**Electronic supplementary material:**

The online version of this article (doi:10.1186/s12885-016-2208-2) contains supplementary material, which is available to authorized users.

## Background

Myelofibrosis (MF), polycythemia vera (PV), and essential thrombocythemia (ET) are Philadelphia chromosome−negative myeloproliferative neoplasms (MPNs) that are frequently associated with the *JAK2*^V617F^ mutation [1]. Presentations and symptom profiles of these MPNs vary with subtype but often include erythrocytosis, thrombocytosis, leukocytosis, and/or splenomegaly [1, 2]. Prevalence of PV and ET is approximately 10 times higher than MF; prevalence per 100,000 residents in the United States (2008–2010) was 4 to 6 people for MF, 45 to 57 for PV, and 39 to 57 for ET [3].

Patients with MPNs experience a broad array of symptoms that negatively affect their quality of life (QoL) [2]. Symptoms often include fatigue, concentration problems, night sweats, pruritus, and splenomegaly-related symptoms (eg, early satiety and abdominal discomfort and/or pain) [4, 5]. In addition, patients with MF, PV, or ET have increased risk of mortality compared with the general population [6]. Cardiovascular events and fibrotic and/or leukemic transformation are important causes of morbidity and mortality in patients with MPNs [7–9]. One study reported that median survival is approximately 6 years for patients with MF (median age at diagnosis, 63 years; median follow-up, 8 years); 14 years for patients with PV (median age at diagnosis, 64 years; median follow-up, 12 years); and 20 years for patients with ET (median age at diagnosis, 55 years; median follow-up, 17 years) [10].

Numerous questions remain regarding myeloproliferative disease burden and management. A limited amount of data about the extent to which MPNs affect activities of daily living are available in the published literature [11], and we are unaware of any published reports about the productivity of patients with MPNs who are employed. Methods for identifying high-risk patients have been developed based on known risk factors [12–14] and symptom severity [15]. Prognostic risk score models have been proposed for MF [12], PV [13], and ET [14]; however, the predictive value of these systems for identifying comprehensive patient burden that includes QoL and productivity impairments has not been evaluated.

The MPN Landmark survey evaluated the patient disease burden in the Philadelphia chromosome–negative MPN disease setting. This first analysis of the MPN Landmark survey includes data concerning the impact of MPNs on health and productivity as reported by a contemporary population of respondents with MPNs in the United States.

## Methods

### Respondents

Patients in the United States with a previous diagnosis of MF, PV, or ET were eligible to take the survey. Respondents were recruited through physician offices, advocacy groups, and the media. Invitations to complete a web-based survey were delivered by direct mail to patients nationwide. Digital recruiting was conducted online at 50 websites and a print ad campaign was conducted across 13 newspapers in 5 major metropolitan regions (Chicago, IL; Dallas, TX; Houston, TX; Philadelphia, PA; and New York, NY). To supplement the multichannel recruitment effort, 1500 additional invitations were distributed through specialists who were treating patients with MPNs. Surveys were administered online and completed between May and July 2014; respondents did not receive remuneration for participating. Investigators were blinded to the method by which individual respondents were recruited, and the survey did not ask respondents to report their recruitment method.

### Survey instrument

A web-based survey that included 65 multiple-choice questions with an estimated completion time of 20 to 25 minutes was presented to each respondent. A summary of the patient respondent portion of the MPN Landmark survey is included in the Additional file 1: Respondents answered questions tailored to their diagnosis—MF, PV, or ET. The current report includes observational findings from respondents based on questions related to (1) respondent demographics; (2) disease features; (3) symptom burden; (4) disease burdens related to QoL, activities of daily living, and work productivity; and (5) treatment management and therapies. Individual MPN-related symptoms were evaluated using an adapted version of the MPN Symptom Assessment Form (MPN-SAF) [5] and were rated on a scale that ranged from 0 (absent) to 10 (worst imaginable); based on the structure of the scale, individual symptoms with severity scores ≥7 were considered very severe. Questions evaluating emotional impact and burden of disease were evaluated on a scale that ranged from 1 (not at all) to 5 (a great deal). Comorbidities were based on respondent answers to questions about “current medical conditions” and were not confirmed with medical records (eg, “leukemia” could represent any leukemia subtype or other blood disorders that respondents correctly or incorrectly considered to be leukemia).

### Statistical methods

Study findings were analyzed using descriptive statistics. The survey included a core set of mandatory questions that required answers before completion. Analyses based on optional questions excluded all respondents who did not provide an answer. Respondent outcomes were examined by respondent-reported symptom severity quartile and calculated prognostic risk scores. To identify trends related to the effect of MPN symptom severity on activities of daily living and work productivity, respondents were stratified by disease-related symptom severity quartile using abbreviated (10-item) MPN-SAF total symptom scores (MPN-SAF TSS) [4]. Previous studies of MPN-SAF TSS quartiles in patients with MPNs found that higher quartiles were associated with increased measures of disease severity (eg, presence of cytopenias, prior thrombosis, individual symptom scores) [15, 16]. On a scale of 0 (absent) to 100 (worst imaginable), symptom severity quartiles were determined after survey data collection in an effort to include similar numbers of respondents in each quartile and were defined as follows: quartile 1, 0–5; quartile 2, 6–13; quartile 3, 14–26; quartile 4, 27–78. Respondents were also stratified by prognostic risk scores to identify trends related to the effects of risk scores on activities of daily living and work productivity. Prognostic risk scores were calculated based on information provided by respondents regarding medical history events and laboratory values at any point between diagnosis and the time of the survey and were generated using published scoring systems for MF (Dynamic International Prognostic Scoring System) [12], PV (modified from Tefferi et al.) [13], and ET (International Prognostic Score for Essential Thrombocythemia) [14] (Additional file 1: Table S1). Respondents who could not recall required information for prognostic risk score calculations were excluded from the related subgroup analyses. This report analyzed quartile 1 and quartile 4 symptom severity subgroups and low- and high-risk prognostic risk score subgroups in an effort to focus on (1) respondents with potentially underappreciated MPN disease severity (ie, those with the lowest symptom severity or low prognostic risk) and (2) respondents with the potentially greatest disease severity (ie, those with the highest symptom severity and/or high prognostic risk).

### Ethics, consent and permissions

The study received approval from the ICF International internal ethics review board, which is registered with the US Department of Health and Human Services Office of Human Research Protections and has Federalwide Assurance (FWA #00000845; ethics review board chair, Janet Griffith, PhD). ICF International assisted in conducting the MPN Landmark survey. All respondents provided informed consent.

## Results

### Respondent demographics

The survey was completed by 813 respondents (MF, *n* = 207; PV, *n* = 380; ET, *n* = 226), representing 47 states and the District of Columbia. Most respondents were women; 98 % were white (Table 1). The median age was similar between groups (MF, 66 years; PV, 64 years; ET, 62 years), and a subgroup of respondents were <50 years of age at diagnosis (MF, 19 %; PV, 27 %; ET, 43 %). Nearly all respondents reported being covered by some form of health insurance, predominantly group commercial insurance or Medicare. A subset of respondents reported that they relied on a caregiver (either “rarely,” “sometimes,” or “often”) because of their MPN (MF, 41 %; PV, 22 %; ET, 15 %).

**Table 1:** Respondent characteristics

	MF (*n* = 207)	PV (*n* = 380)	ET (*n* = 226)
Median (range) age, y	66 (28–90)	64 (25–94)	62 (23–87)
Women, n (%)	112 (54)	237 (62)	163 (72)
Race,^a^ n (%)			
White	203 (98)	371 (98)	221 (98)
Black or African American	2 (1)	3 (1)	2 (1)
Asian	1 (<1)	4 (1)	2 (1)
Unknown	1 (<1)	2 (1)	1 (<1)
Median (range) disease duration since diagnosis, y	4 (0–36)	7 (0–61)	7 (0–36)
Calculated prognostic risk score, n (%)			
Low	9 (4)	26 (7)	46 (20)
Intermediate-1	34 (16)	77 (20)	86 (38)
Intermediate-2	84 (41)	62 (16)	NA^b^
High	63 (30)	101 (27)	35 (16)
Missing^c^	17 (8)	114 (30)	59 (26)
Primary medical insurance, n (%)			
Medicare	94 (45)	157 (41)	84 (38)
Group commercial insurance	80 (39)	169 (45)	103 (46)
Individual commercial insurance	14 (7)	25 (7)	18 (8)
Medicaid or state assistance	1 (<1)	3 (1)	1 (<1)
Tricare or VA benefit	6 (3)	4 (1)	8 (4)
Medicare and group insurance through current/former employer	3 (1)	3 (1)	2 (1)
Medicare and secondary/supplemental insurance	3 (1)	2 (1)	2 (1)
Other	5 (2)	13 (3)	5 (2)
Unknown	1 (<1)	2 (1)	0
Education, n (%)			
No high school	1 (<1)	0	0
Some high school	0	1 (<1)	1 (<1)
High school graduate	17 (8)	33 (9)	20 (9)
Technical postsecondary	7 (3)	11 (3)	11 (5)
Some college	48 (23)	88 (23)	63 (28)
Four-year college graduate	67 (32)	116 (31)	58 (26)
Postgraduate degree	67 (32)	131 (35)	73 (32)
Household income, n (%)			
≤$15,000	6 (3)	12 (3)	9 (4)
15,001–$25,000	9 (4)	21 (6)	9 (4)
25,001–$35,000	19 (9)	22 (6)	11 (5)
35,001–$50,000	17 (8)	37 (10)	22 (10)
50,001–$75,000	34 (16)	62 (16)	35 (16)
75,001–$100,000	47 (23)	53 (14)	60 (27)
>$100,000	62 (30)	145 (38)	67 (30)
Unknown	13 (6)	28 (7)	13 (6)

ET = essential thrombocythemia; MF = myelofibrosis; NA = not applicable; PV = polycythemia vera; VA = Veterans Affairs

^a^Respondents were allowed to give multiple answers regarding race; this table records only the first answer given by each respondent

^b^Respondents with ET could receive prognostic risk scores of low, intermediate, or high; intermediate was not divided into intermediate-1 and -2

^c^Calculated prognostic risk score missing because of unknown lab values for risk categorization

### Disease features

Median disease durations for respondents with MF, PV, and ET were 4 years, 7 years, and 7 years, respectively (Table 1). Most respondents had an intermediate or high prognostic risk score calculated using information collected during the survey and previously published scoring systems described in Additional file 1: Table S1.

A subgroup of respondents had comorbidities at the time of the survey (MF, 44 %; PV, 37 %; ET, 37 %). The most frequently reported comorbidities for respondents with MF were diabetes (6 %), moderate to severe kidney disease (6 %), emphysema/chronic obstructive pulmonary disease (COPD)/chronic bronchitis (5 %), and leukemia (5 %); for respondents with PV, diabetes (7 %), connective tissue disorders (6 %), moderate to severe kidney disease (5 %), and emphysema/COPD/chronic bronchitis (5 %); and for respondents with ET, moderate to severe kidney disease (5 %), myocardial infarction (4 %), diabetes (4 %), solid tumor (4 %), and narrowing and hardening of the arteries to the limbs (4 %).

### Symptom burden

In agreement with other reports in patients with MPNs [4, 5, 18], the MPN Landmark survey respondent population reported a broad symptom burden; Table 2 presents the mean scores and incidences of symptoms included in the MPN-SAF TSS for respondents who experienced symptoms within the last 12 months. Among respondents with MF, the mean MPN-SAF TSS was more severe in those with higher versus lower calculated prognostic risk scores (MPN-SAF TSS in highest vs lowest prognostic risk category, 30.8 vs 8.1). However, this trend was not observed among respondents with PV (MPN-SAF TSS in highest vs lowest prognostic risk category, 16.2 vs 16.8) or ET (13.1 vs 18.1, respectively). A subset of respondents reported that their symptoms were very severe (ie, severity score of ≥7 out of 10; Additional file 1: Fig. S1). Most respondents had symptoms at diagnosis (MF, 78 %; PV, 88 %; ET, 81 %), with fatigue being the most frequently reported.

**Table 2:** MPN-SAF 10-item symptoms reported by MPN within the last 12 months^a^

	MF (*n* = 207)		PV (*n* = 380)		ET (*n* = 226)	
Symptom	Mean Score	Incidence, %	Mean Score	Incidence, %	Mean Score	Incidence, %
Fatigue^b^	5.1	80	4.6	73	4.3	71
Abdominal discomfort	2.3	53	1.4	35	1.3	31
Night sweats^c^	2.3	51	2.0	45	1.9	38
Early satiety	2.0	37	1.1	22	1.0	21
Bone pain^d^	2.0	40	1.2	23	1.2	27
Inactivity	1.9	31	1.5	24	1.0	19
Itching	1.8	40	2.9	55	1.4	33
Weight loss	1.6	28	0.5	12	0.5	10
Concentration problems	1.5	29	2.0	36	1.9	35
Fever	0.6	14	0.2	5	0.2	6
MPN-SAF TSS	21.2	NA	17.4	NA	14.8	NA

ET = essential thrombocythemia; MF = myelofibrosis; MPN = myeloproliferative neoplasm; MPN-SAF TSS = Myeloproliferative Neoplasm Symptom Assessment Form 10-item total symptom score; NA = not applicable; PV = polycythemia vera

^a^This table summarizes only those symptoms included in the MPN-SAF 10-item instrument and is not inclusive of all symptoms that were assessed in the MPN Landmark survey. Symptom severity score was on a scale of 0 (absent) to 10 (worst imaginable); mean scores included in this table were calculated among those respondents who reported experiencing the symptom (ie, score ≥1) within the 12 months preceding the survey

^b^Presented as “fatigue” to respondents with MF and PV and as “fatigue and tiredness” to respondents with ET

^c^Presented as “night sweats” to respondents with MF and ET and as “day and night sweats” to respondents with PV

^d^Diffuse, not joint pain or arthritis

Many respondents reported that ≥1 MPN-related symptom manifested ≥1 year before diagnosis (MF, 49 %; PV, 61 %; ET, 58 %). Notable proportions of respondents with MF reported that fatigue (29 %) and difficulty sleeping (15 %) manifested ≥1 year before diagnosis. Respondents with PV and ET reported that the most common symptoms to manifest ≥1 year before diagnosis were fatigue (26 % and 23 %, respectively) and headaches (16 % and 16 %).

### Quality of life, activities of daily living, and work productivity

**Fig. 1:**
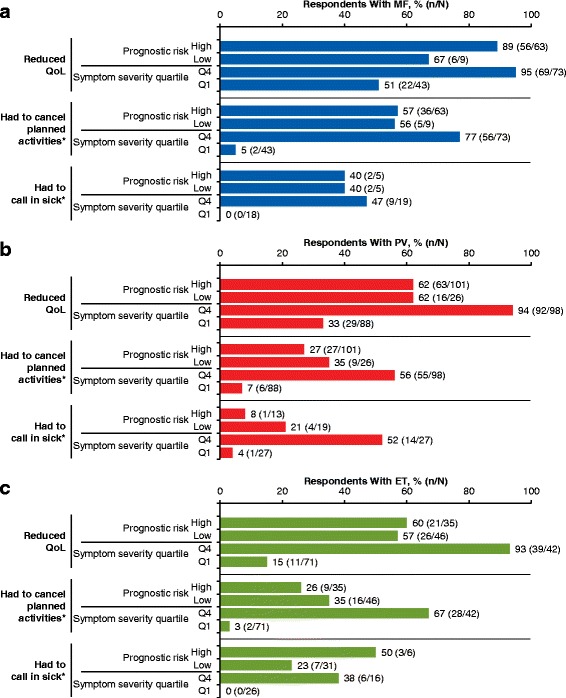
Impact of MPNs on QoL, work, and activities of daily living. MPN impact was stratified by calculated prognostic risk score and symptom severity quartile in respondents with (**a**) MF, (**b**) PV, and (**c**) ET. ET = essential thrombocythemia; MF = myelofibrosis; MPN = myeloproliferative neoplasm; PV = polycythemia vera; Q1 = quartile 1; Q4 = quartile 4; QoL = quality of life. * ≥ 1 day in the preceding 30 days

Many respondents reported that their MPN-related symptoms reduced their QoL (MF, 81 %; PV, 66 %; ET, 57 %). Reduced QoL due to MPN-related symptoms was self-reported even by respondents with low calculated prognostic risk scores (MF, 67 %; PV, 62 %; ET, 57 %) and those in the lowest symptom severity quartile (MF, 51 %; PV, 33 %; ET, 15 %; Fig. 1).

A notable proportion of respondents reported that their MPN interfered with activities of daily living (Table 3). Many respondents (≥45 %) in each group reported that their activities were limited by pain/discomfort; some respondents (MF, 12 %; PV, 10 %; ET, 7 %) reported that this occurred “a great deal” (Table 3). More than 10 % of respondents in each group reported that their MPN caused the cancelation of planned activities in ≥4 of the preceding 30 days (Table 3). Among respondents with low prognostic risk scores, ≥35 % reported canceling ≥1 day of planned activities in the preceding 30 days because of their condition (Fig. 1). Most respondents reported feeling anxious or worried about their MPN (MF, 91 %; PV, 78 %; ET, 74 %) and 63 % of respondents with PV reported stress/anxiety related to managing their hematocrit at <45 %. Many respondents reported feeling depressed (MF, 75 %; PV, 60 %; ET, 59 %) and/or angry (MF, 43 %; PV, 38 %; ET, 38 %). Some respondents had difficulty coping with stress (MF, 50 %; PV, 46 %; ET, 43 %). Respondents also reported altered sleeping habits (MF, 57 %; PV, 53 %; ET, 47 %). In addition, many respondents felt that their MPN affected their family/social life (MF, 79 %; PV, 63 %; ET, 55 %), relationship with their caregiver (MF, 28 %; PV, 18 %; ET, 15 %), and sex life (MF, 63 %; PV, 49 %; ET, 42 %).

**Table 3:** MPN effect on activities of daily living

Effect on Activities, n (%)	MF (*n* = 207)	PV (*n* = 380)	ET (*n* = 226)
Interfered with daily activities^a^			
At all^b^	110 (53)	181 (48)	84 (37)
A great deal^c^	43 (21)	36 (10)	15 (7)
Interfered with family or social life^a^			
At all^b^	163 (79)	241 (63)	125 (55)
A great deal^c^	35 (17)	43 (11)	18 (8)
Activities limited by pain/discomfort^a^			
At all^b^	127 (61)	197 (52)	101 (45)
A great deal^c^	25 (12)	36 (10)	15 (7)
Days canceling planned activities^d^			
1–3	44 (21)	68 (18)	41 (18)
4–6	22 (11)	26 (7)	14 (6)
7–9	2 (1)	0	3 (1)
10–12	7 (3)	9 (2)	4 (2)
13–15	3 (1)	2 (1)	1 (<1)
≥16	8 (4)	6 (2)	1 (<1)
Days spent in bed (all or most of the day)^d^			
1–3	38 (18)	54 (14)	30 (13)
4–6	13 (6)	15 (4)	10 (4)
7–9	1 (<1)	4 (1)	5 (2)
10–12	4 (2)	2 (1)	5 (2)
13–15	4 (2)	5 (1)	1 (<1)
≥16	7 (3)	9 (2)	5 (2)

ET = essential thrombocythemia; MF = myelofibrosis; MPN = myeloproliferative neoplasm; PV = polycythemia vera

^a^Ever

^b^A score >1 on a scale of 1 (not at all) to 5 (a great deal)

^c^A score of 5 on a scale of 1 (not at all) to 5 (a great deal)

^d^In the preceding 30 days

Many respondents reported that their MPN limited productivity, including reduced work hours, calling in sick to work, and/or terminating their job (Table 4). Even respondents with low calculated prognostic risk scores reported calling in sick to work at least once in the preceding 30 days (Fig. 1). However, a consistent trend with regard to productivity and calculated prognostic risk scores was not observed across all 3 MPN subgroups. Greater proportions of respondents with PV who had low prognostic risk scores reported canceling planned activities and calling in sick to work compared with those who had high prognostic risk scores. Respondents in the high symptom severity quartile of all 3 MPN subgroups called in sick to work more often than respondents in the low symptom severity quartile.

**Table 4:** Effect of MPNs on work and productivity among respondents who were employed

Effect on Work/Productivity,^a^ n/N^b^ (%)	MF (*n* = 207)	PV (*n* = 380)	ET (*n* = 226)
Reduced work hours^c^	70/119 (59)	91/246 (37)	50/169 (30)
Days sick from work^d^			
1–3	11/52 (21)	19/127 (15)	18/88 (20)
4–6	3/52 (6)	4/127 (3)	2/88 (2)
7–9	0	0	0
≥10	1/52 (2)	1/127 (1)	0
Voluntarily terminated job^c^	39/125 (31)	54/254 (21)	19/169 (11)
Involuntarily terminated job^c^	6/120 (5)	11/242 (5)	7/168 (4)
Medical disability^c^	38/134 (28)	37/253 (15)	12/177 (7)
Early retirement^c^	38/125 (30)	54/253 (21)	24/169 (14)

ET = essential thrombocythemia; MF = myelofibrosis; MPN = myeloproliferative neoplasm; PV = polycythemia vera

^a^Respondents employed full- or part-time only

^b^N is the number of respondents who reported “yes” or “no,” excluding those who answered “not applicable.”

^c^Ever

^d^In the preceding 30 days

### Treatment management and therapies

The most important treatment goal reported by respondents with MF (42 %) or PV (25 %) was slowing or delaying progression of their disease; prevention of vascular/thrombotic events was the most important treatment goal reported by respondents with ET (35 %) (Table 5). A subset of respondents reported symptom relief as their most important treatment goal (MF, 7 %; PV, 9 %; ET, 9 %). In all MPN groups, fatigue was the most common symptom that respondents reported as the one they most wanted to resolve (MF, 47 %; PV, 33 %; ET, 33 %).

**Table 5:** Most important treatment goals

		Symptom Quartile		Prognostic Risk	
Treatment Goal,^a^ n (%)	All Respondents	Q4	Q1	High	Low
MF	*n* = *207*	*n* = *73*	*n* = *43*	*n* = *63*	*n = 9*
Slow/delay progression of condition	86 (42)	27 (37)	18 (42)	28 (44)	6 (67)
Better QoL	43 (21)	26 (36)	2 (5)	16 (25)	0
Healthy blood counts	23 (11)	6 (8)	10 (23)	5 (8)	1 (11)
Symptom improvement	14 (7)	5 (7)	1 (2)	1 (2)	1 (11)
Reduction in spleen size	13 (6)	2 (3)	5 (12)	3 (5)	0
Reduce blood transfusions	12 (6)	4 (6)	2 (5)	6 (10)	0
PV	*n = 380*	*n = 98*	*n = 88*	*n = 101*	*n = 26*
Slow/delay progression of condition	96 (25)	23 (24)	21 (24)	20 (20)	7 (27)
Prevention of vascular/thrombotic events	92 (24)	17 (17)	16 (18)	25 (25)	5 (19)
Healthy blood counts	68 (18)	10 (10)	27 (31)	22 (22)	3 (12)
Better QoL	45 (12)	19 (19)	5 (6)	11 (11)	4 (15)
Symptom improvement	33 (9)	21 (21)	1 (1)	6 (6)	2 (8)
Hematocrit levels <45 %	22 (6)	4 (4)	9 (10)	9 (9)	1 (4)
ET	*n = 226*	*n = 42*	*n = 71*	*n = 35*	*n = 46*
Prevention of vascular/thrombotic events	78 (35)	9 (21)	28 (39)	12 (34)	15 (33)
Slow/delay progression of condition	48 (21)	8 (19)	16 (23)	5 (14)	14 (30)
Healthy blood counts	39 (17)	5 (12)	18 (25)	5 (14)	4 (9)
Better QoL	31 (14)	14 (33)	2 (3)	3 (9)	9 (20)
Symptom improvement	21 (9)	3 (7)	3 (4)	6 (17)	2 (4)

ET = essential thrombocythemia; MF = myelofibrosis; PV = polycythemia vera; QoL = quality of life

^a^Most important treatment goal, other than a cure, reported by ≥5 % of respondents in each MPN group

The therapies that respondents most often reported receiving at any time were aspirin (59 %), ruxolitinib (48 %), and hydroxyurea (42 %) in the MF group; phlebotomy (90 %), aspirin (83 %), and hydroxyurea (58 %) in the PV group; and aspirin (87 %), hydroxyurea (69 %), and anagrelide (36 %) in the ET group.

## Discussion

The MPN Landmark survey is the first large survey to evaluate the experience of patients with MPNs in a contemporary US population and is the first study to extensively evaluate effects of MPNs on productivity and employment. The survey findings suggest that patients with MPNs experience a broad symptom burden and reductions in QoL, functional status, activities of daily living, and work productivity. These findings support recent reports of symptom burden and QoL that included non-US patient populations [4, 5, 17].

Increased recognition of the full disease burden associated with MPNs will help identify patients with unmet needs who may benefit from a change in management and is an important step toward improving patient care. Findings of this survey suggest that prognostic risk score may not capture all aspects of MPN disease burden. Notably, respondents with low prognostic risk scores reported experiencing disease burdens that may be underreported and underappreciated, highlighting an unmet need among patients with low prognostic risk scores. Mean MPN-SAF TSS values were actually higher (ie, more severe) in respondents with PV or ET who had low prognostic risk scores compared with those who had high risk scores. This discordance between prognostic risk score and MPN-SAF TSS was not observed in respondents with MF and may be explained by the inclusion of constitutional symptoms in the risk category calculation for MF but not PV or ET (Additional file 1: Table S1).

Symptoms related to MPNs are informative for early diagnosis and assessing patient clinical needs, but it is not uncommon for patients to experience symptoms well in advance of a formal diagnosis. Nearly one half of respondents with MF and the majority of respondents with PV or ET in the MPN Landmark survey reported experiencing MPN-related symptoms ≥1 year before diagnosis. The MPN Landmark survey also provided new and important data regarding the negative effects of MPNs on activities of daily living and work productivity and indicated that respondents with the most severe symptoms (ie, the highest symptom severity quartile) more frequently reported negative effects on QoL, productivity, and activities of daily living compared with the lowest quartile. In contrast, prognostic risk score was not consistently correlated with these measures of QoL and functionality. Improvements in symptom recognition and treatment may help ameliorate these negative effects.

Patient care in the MPN setting may be improved with updated management strategies. This study highlights the importance of using surveys or questionnaires—such as the MPN-SAF, the Cancer Support Source™ distress screening tool [18], or similar systematic approaches—to accurately capture patient-reported disease burden on a regular basis. Furthermore, participation in registries, such as the Cancer Experience Registry [19], may help communicate general patient symptoms and unmet needs to the broader field. Some symptomatic patients, including those with low prognostic risk scores, may benefit from a change in treatment. It will be important for physicians and researchers to optimize prognostic tools for identifying such patients and to evaluate potential biomarkers that could be used for making a targeted treatment change. For example, serum cytokines may be informative biomarkers for patients with MPNs. Levels of several serum cytokines are altered in patients with MPNs and have been correlated with disease characteristics, including symptoms and survival [20–22]. Further work will be required to validate these findings and determine if and how serum cytokine levels or other potential biomarkers could be used in clinical practice.

Limitations of the study were primarily a result of the descriptive design, self-reported nature of the survey, variations in respondent demographics, and challenges related to the relatively low prevalence of MPNs. The study was designed to be analyzed descriptively, which precluded statistical comparisons of the data. Because all results were self-reported by patient respondents, including treatments and risk factors used in the calculation of prognostic risk scores, data concerning symptom severity, outcomes, and comorbidities were not confirmed with clinical measures or respondents’ treating physicians. In addition, the sampling procedures may have introduced self-selection biases that could have affected the demographics of the respondents who participated. For example, relatively few low-risk respondents with MF or PV completed the survey; it remains unclear if this accurately represents the MPN population in the United States or if more severely affected patients with MF and PV were more motivated to participate. Respondents were predominantly college educated, with a mean annual household income > $75,000, compared with the median US household income in 2013, which was $52,250 [23]. In addition, although some symptom- and QoL-related questions were adapted from the validated MPN-SAF [5] and European Organisation for Research and Treatment of Cancer Quality of Life Questionnaire − Core 30 [24] instruments, the MPN Landmark survey itself did not include use of validated QoL instruments. As a result, the MPN Landmark survey may underrepresent the symptom burden experienced by the general MPN patient population. Because patients with MPNs are somewhat rare, the sample needed to be recruited by nonprobability sampling methods, which restricted the use of probability statistics to generate sample estimates. Notwithstanding these limitations, this was the only feasible methodology for assessing these rare conditions in a nationally distributed general population sample.

## Conclusions

In conclusion, the MPN Landmark survey is the first large survey of its kind. As may be expected, patient respondents indicated that their most important treatment goals were to slow/delay disease progression and to prevent vascular/thrombotic events. However, the data also suggest that the disease burden experienced by patients with MPNs in the United States has been underreported in the literature and negatively affects QoL, activities of daily living, and the ability to work and be productive, including in patients with low prognostic risk scores and low symptom burden. MPN treatment considerations should include reducing the symptom burden as well as improving QoL and productivity to enhance the overall health and lives of patients with MPNs.

## Additional file

Additional file 1: Table S1. Prognostic Risk Scoring Systems. **Figure S1.** Proportion of Respondents Who Reported Individual Symptoms to be Very Severe. (PDF 140 kb)

## Abbreviations

COPD: chronic obstructive pulmonary disease
ET: essential thrombocythemia
MF: myelofibrosis
MPN: myeloproliferative neoplasm
MPN-SAF: Myeloproliferative Neoplasm Symptom Assessment Form
MPN-SAF TSS: Myeloproliferative Neoplasm Symptom Assessment Form 10-item total symptom score
PV: polycythemia vera
QoL: quality of life
VA: Veterans Affairs
