# Supplementary material for: Myeloproliferative neoplasms (MPNs) have a significant impact on patients’ overall health and productivity: the MPN Landmark survey
Source: BMC Cancer. 2016 Feb 27;16:167. doi: 10.1186/s12885-016-2208-2 (PMC4769833; doi:10.1186/s12885-016-2208-2)
Supplement: Additional file 1: Table S1. — Prognostic Risk Scoring Systems. Figure S1. Proportion of Respondents Who Reported Individual Symptoms to be Very Severe. (PDF 140 kb) [file 12885_2016_2208_MOESM1_ESM.pdf]

Myeloproliferative Neoplasms (MPNs) Have a Significant Impact on Patients' Overall Health and Productivity: The MPN Landmark Survey

Ruben Mesa, MD,[1] Carole B. Miller, MD,[2] Maureen Thyne, PA,[3] James Mangan, MD, PhD,[4] Sara Goldberger, LCSW-R,[5] Salman Fazal, MD,[6] Xiaomei Ma, PhD,[7] Wendy Wilson, RN, MSN, OCN,[8] Dilan C. Paranagama, PhD,[9] David G. Dubinski, MBA,[9] John Boyle, PhD,[10] John O. Mascarenhas, MD, MS[11]

[1]Mayo Clinic Cancer Center, Scottsdale, AZ; [2]St. Agnes Hospital, Baltimore, MD; [3]Weill Cornell Medical College, New York, NY; [4]University of Pennsylvania, Philadelphia, PA; [5]Cancer Support Community, New York, NY; [6]Allegheny Health Network, Pittsburgh, PA; [7]Yale School of Public Health, New Haven, CT; [8]Fred Hutchinson Cancer Research Center, Seattle, WA; [9]Incyte Corporation, Wilmington, DE; [10]ICF International, Fairfax, VA; [11]Icahn School of Medicine at Mount Sinai, New York, NY

Appendix

Table of Contents

Supplementary Tables 2
Supplementary Figures 3
MPN Landmark Survey: Patient Survey 5

SUPPLEMENTARY TABLES

Supplementary Table 1. Prognostic Risk Scoring Systems

	MF — DIPSS		PV		ET — IPSET	
	**Factor**	**Points**	**Factor**	**Points**	**Factor**	**Points**
**Source reference**	Passamonti et al 2010 [12]		Tefferi et al 2013 [13]		Passamonti et al 2012 [14]	
**Age, y**	>65	1	≥67	5	≥60	2
	≤65	0	57–66	2	<60	0
			<57	0		
**Leukocyte count, × 10^9/L**	>25	1	≥15	1	≥11	1
	≤25	0	<15	0	<11	0
**Hemoglobin, g/dL**	<10	2	NA		NA	
	≥10	0				
**Constitutional symptoms***	Present	1	NA		NA	
	Absent	0				
**Peripheral blood blasts, %**	≥1	1	NA		NA	
	<1	0				
**Prior Thrombosis**	NA		Yes	1	Yes	1
			No	0	No	0
**Prognostic risk group, score**						
**Low**	0		0		0	
**Intermediate-1**	1–2		1–2		1–2†	
**Intermediate-2**	3–4		3			
**High**	≥5		≥4		3–4	

DIPSS=Dynamic International Prognostic Scoring System; ET=essential thrombocythemia; IPSET=International Prognostic Score for ET; MF=myelofibrosis; NA=not applicable; PV=polycythemia vera.
*Greater than 10% weight loss in the last 6 months, night sweats, or unexplained fever >37.5°C.
†Respondents with ET could receive prognostic risk scores of low, intermediate, or high; intermediate was not divided into intermediate-1 and -2.

SUPPLEMENTARY FIGURES

Supplementary Figure 1. Proportion of respondents who reported individual symptoms to be very severe. Very severe was defined as a symptom score of ≥7. Only respondents with a history of each symptom were included in this analysis; respondents who reported a score of 0 (ie, symptom absent) were excluded. ET=essential thrombocythemia; MF=myelofibrosis; PV=polycythemia vera. *Presented as "fatigue" to respondents with MF and PV and as "fatigue and tiredness" to respondents with ET. [†]Diffuse, not joint pain or arthritis. [‡]Presented as "night sweats" to respondents with MF and ET and as "day and night sweats" to respondents with PV.

Supplementary Figure 1. Proportion of Respondents Who Reported Individual Symptoms to be Very Severe

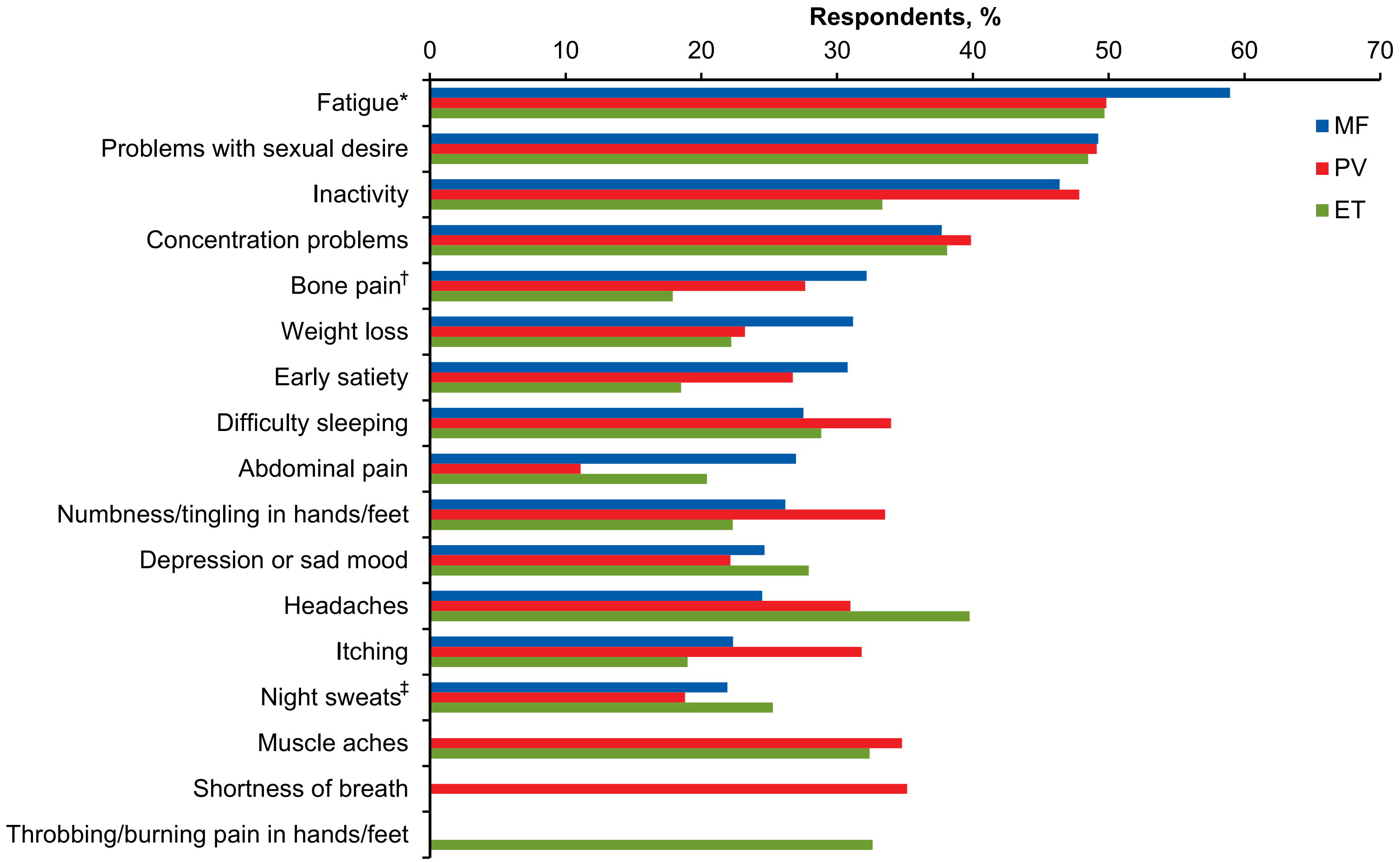

MPN LANDMARK SURVEY: PATIENT SURVEY

Summary of 65-item Questionnaire

The LANDMARK MPN patients' survey was a web-based questionnaire with both qualitative and quantitative questions for which patients were asked to assess health status, disease severity, care, and treatment satisfaction by Likert-like scales and multiple choice answers. Patients were asked the following questions:

Q1. How old are you?
Q2-4. What is your current diagnosis? At what age or what year were you first diagnosed?
Q5. Do you live in the USA?
Q6. In what state do you live?
Q7. How would you describe your current state of health?
Q8. Have you ever had any of the following symptoms?

- Abdominal discomfort
- Abdominal pain
- Blood clot(s)
- Bone pain (diffuse not joint pain or arthritis)
- Bruising
- Cough
- Day or night sweats
- Depression or sad mood
- Difficulty sleeping
- Dizziness/vertigo/lightheadedness
- Double or blurred vision
- Facial flushing
- Fatigue
- Fatigue or tiredness
- Fever (>100°F)
- Filling up quickly when you eat (early satiety)
- Hypertension
- Inactivity
- Itching (pruritus)
- Muscle aches
- Night sweats
- Nosebleeds
- Numbness/singling in my hands and feet
- Problems with concentration
- Problems with headaches
- Problems with sexual desire
- Redness, throbbing and burning pain in the hands and feet
- Shortness of breath
- Stroke
- Unintentional weight loss
- Vision changes
- Weakness
- None

Q9. Which of these symptoms were you experiencing at time of diagnosis?
Q10. How long before you were diagnosed with MF/PV/ET did you first experience any of these symptoms?
Q12. Which of these symptoms do you feel are the result of your disease?
Q13. How severe are the following symptoms on a scale from 0 (absent) to 10 (worst imaginable) as experienced in the last 12 months?
Q14. Of the symptoms that you are currently experiencing, which **one** would you most like to resolve?

Q15. What is the next (2nd) symptom you would like to most resolve?
Q16. What is the next (3rd) symptom you would most like to resolve?
Q17. In the last 30 days, how many days did you have to cancel planned or scheduled activities (i.e., lunch with friends, shopping trip, appointment, etc.) as a result of your disease?
Q18. In the last 30 days, how many days did you stay in bed all or most of the day as a result of your disease?
Q19. Do you currently have any of the following conditions?

- Heart attack (myocardial infarction)
- Congestive heart failure
- Narrowing and hardening of the arteries to the limbs (peripheral vascular disease)
- Narrowing and hardening of the arteries to the brain (cerebrovascular disease)
- Dementia
- Emphysema/COPD/chronic bronchitis
- Connective tissue disorders (lupus, rheumatoid arthritis, psoriasis, etc.)
- Peptic ulcer disease
- Diabetes
- Moderate to severe kidney disease
- Leukemia
- Malignant lymphoma
- Solid tumor (i.e. breast cancer, lung cancer, prostate cancer, or other non blood cancer)
- Liver disease
- AIDS
- Other (specify)
- None

Q20. Do you rely on a caregiver to help you with your condition, if so who is your main caregiver?
Q21. As a result of your disease have you ever:

- Reduced your hours at work
- Voluntarily terminated your job
- Been involuntarily terminated from your job
- Gone on medical disability
- Taken early retirement

Q22. What is your current employment status?
Q23. In the last 30 days, how many days did you have to call in sick as a result of your symptoms?
Q24. Rank the following statements (on a scale from 1 [not at all] to 5 [a great deal]) as they have occurred during the past month, as a result of your disease:

- I have felt anxious or worried about my condition
- I have felt depressed or discouraged
- I have been irritable or unusually angry and I have not controlled it well
- My sleeping habits have changed
- I have noticed a change in my appetite
- I have had trouble focusing at work or at home, or on routine things such as reading the newspaper or watching television
- My condition has caused physical, emotional or financial hardship for me
- My condition has caused changes in how I look, and this concerns me

- I have had trouble coping with the stress I have been having
- My condition is controlling my life
- I have avoided social interaction with others due to physical embarrassment of my appearance because of my condition

Q25. To what extent (on a scale from 1 [not at all] to 5 [a great deal]) does MF/PV/ET interfere with the following activities in your life?

- Daily activities
- Family or social life
- Sex life
- Pain and discomfort have caused me to limit my activities
- Relationship with my caregiver

Q26. Did you ever suffer from any of the following prior to being diagnosed with MF/PV/ET?

Mark all that apply:

- Easy bruising
- Nosebleeds
- Heavy menstrual periods
- Bleeding ulcer
- Esophageal bleeding
- Rectal bleeding
- Blood in urine
- Other (specify)

Q27. Were you ever diagnosed with any of the following prior to being diagnosed with MF/PV/ET?

Mark all that apply:

- Stroke
- Transient ischemic attack (TIA)
- Heart attack
- Blood clot in abdomen
- Deep vein thrombosis (DVT)
- Blood clot in lung (pulmonary embolus)
- Other (specify)

Q27Lab. (Q27a) Since you were diagnosed with MF/PV/ET, have your lab results ever shown any of the following factors?

Mark all that apply:

- Anemia (hemoglobin levels below 10 g/dL)
- MF: Elevated levels of white blood cells (leukocytes) greater than 25×10^9/L
- PV: Elevated levels of white blood cells (leukocytes) greater than 15×10^9/L
- ET: Elevated levels of white blood cells (leukocytes) greater than 11×10^9/L
- Elevated levels of blasts (immature blood cells) above 1% in peripheral blood smear
- Don't know
- None of the above

Q28. Select any of the following treatments that you have ever received to help manage your MF/PV/ET:

Mark all that apply:

- Aspirin
- Allegra, Claritin, Zyrtec or other antihistamines
- Bone marrow transplant or stem cell transplant <<if yes, ask Q28cc>>
- PV/ET: Phlebotomy <<if yes, ask Q28dd>>
- Removal of spleen <<if yes, ask Q28ee>>
- Lexapro, Remeron, Zoloft or other antidepressants
- Prednisone or other anabolic steroids
- Aristocort, Entocort, Medrol or other corticosteroids

- Aranesp, Epogen, Procrit (epoetin alfa injection)
- Droxia, Hydrea (hydroxyurea)
- Jakafi (ruxolitinib)
- Revlimid (lenalidomide)
- Thalomid (thalidomide)
- Coumadin, Heparin, Warfarin (anti-coagulants/blood thinners)
- Pegasys, Peg-Intron (interferon)
- Agrylin (anagrelide)
- MF: Transfusion <<if yes, ask Q28qq>>
- Radiation therapy <<if yes, ask Q28cc>>
- Investigational drug from clinical trial

Q28cc. On a scale from 1 (not at all) to 5 (a great deal), to what extent do side effects from your bone marrow transplant or stem cell transplant treatment have a negative impact on your quality of life?

Q28dd. On a scale from 1 (not at all) to 5 (a great deal), to what extent do side effects from your phlebotomy treatment have a negative impact on your quality of life?

Q28ee. On a scale from 1 (not at all) to 5 (a great deal), to what extent do side effects from removal of spleen have a negative impact on your quality of life?

Q28qq. On a scale from 1 (not at all) to 5 (a great deal), to what extent do side effects from transfusion have a negative impact on your quality of life?

Q28rr. On a scale from 1 (not at all) to 5 (a great deal), to what extent do side effects from radiation therapy have a negative impact on your quality of life?

Q29a. Do you continue to receive phlebotomy treatment to manage your PV?

Q29b. If no, why are you no longer receiving phlebotomy treatments to manage your PV?

- Doctor said it was no longer necessary
- Frequency of visits were inconvenient
- Felt worse after phlebotomy
- Treatment was painful
- Fear of needles
- Other (specify)

Q29c. Have you received a phlebotomy treatment in the last 3 months?

Q29d. How often do you currently receive phlebotomy treatments?

- Every day
- Every other day
- Twice a week
- Once a week
- Once every month
- Once every other month
- Every 3 to 4 months
- Once a year
- Other (specify)

Q29e. In general, how satisfied (very dissatisfied, somewhat dissatisfied, somewhat satisfied, very satisfied, don't know) are you with your phlebotomy treatments?

Q29f. On a scale from 1 (not at all) to 5 (a great deal), to what extent does phlebotomy treatment have a burden on your overall quality of life?

Q29g. On a scale from 1 (not at all) to 5 (a great deal), to what extent do you feel stress/anxiety from managing your hematocrit levels to less than 45%?

Q29h. Do you continue to receive Droxia or Hydrea (hydroxyurea) to manage your PV?

Q29i. In general, how satisfied (very dissatisfied, somewhat dissatisfied, somewhat satisfied, very satisfied, don't know) are you with your Droxia or Hydrea (hydroxyurea) medication?

Q29j. If no longer taking Droxia or Hydrea (hydroxyurea), why?
- Doctor said I was resistant to the medication
- Experienced toxicity to the medication
- Other (specify)
- Don't know

Q30. What measures do you use to determine whether a MF/PV/ET treatment is successful or not successful?
Mark all that apply:
- Feedback from doctor
- Lab results/blood count levels
- Prevention or reduction of thrombotic events
- Reduction in size of spleen
- Fewer symptoms
- PV/ET: Reduce frequency of phlebotomy treatments
- Overall symptom relief
- Enhances my quality of life
- MF: Reduce blood transfusions
- Other (specify)
- None

Q31. Aside from prescription drugs and treatments, select any of the following things you do to manage your MF/PV/ET symptoms?
Mark all that apply:
- Exercise
- Yoga
- Massage
- Mediation
- Acupuncture
- Change in diet to combat specific nutrient deficiencies (i.e., iron)
- Nutritional supplements (Ensure, Boost, etc.)
- Non-prescription supplements (energy supplements, vitamins, herbal supplements)

Q32. Other than a cure for your MF/PV/ET, what is your most important treatment goal for therapy?
- Symptom improvement
- Prevention of vascular/thrombotic events
- MF: Anemia treatment
- MF: Reduce blood transfusions
- Healthy blood counts
- PV/ET: Reduce frequency of phlebotomy treatments
- PV/ET: Hematocrit levels less than 45%
- Reduction in spleen size
- Slow/delay progression of condition
- Better quality of life
- Other (specify)

Q33. What is your next (2ND) most important treatment goal for therapy?

Q34. What is your next (3RD) most important treatment goal for therapy?

Q35. Please indicate whether you agree strongly, agree somewhat, disagree somewhat, or disagree strongly with the following statements:
Q35a. MF/PV/ET symptoms reduce my quality of life
Q35b. MF/PV/ET is a serious health condition

Q35c. MF/PV/ET may progress to more serious conditions
Q35d. PV/ET may increase my risk of heart attacks or strokes

Q36. What is the medical specialty of the doctor that you see most often for your MF/PV/ET?
Select one:
- General/family practice/internal medicine (primary care doctor)
- Hematologist (blood specialist)
- Oncologist (cancer specialist)
- Other (specify)

Q37. How would you describe the setting where you see this doctor most often for your MF/PV/ET?
- Large hospital outpatient clinic
- Small community hospital outpatient clinic
- Solo practice
- Group practice
- Other (specify)

Q38. How many times have you seen your MF/PV/ET doctor in the last 12 months? <<range=0−365>>

Q39. Have you ever changed your MF/PV/ET doctor?

Q39a. Why did you make a change?
- My doctor retired
- My insurance coverage changed
- I relocated to a different city or state
- I was unhappy with the care received
- Other (specify)

Q40. Did your MF/PV/ET doctor classify your current condition with a particular risk score?(For example: High, Intermediate, Low)

Q40a. What is it, if you remember?

Q41. How does the doctor you see most often for your MF/PV/ET assess any symptoms you may be experiencing?
- They listen and wait for me to tell them about any symptoms
- They proactively ask me how I am feeling
- They specifically ask me about most important symptoms
- They ask me to fill a symptom check list and then review each symptom
- They don't ask me about my symptoms
- They don't seem interested in my symptoms

Q42. Thinking about the doctor you see most often for your MF/PV/ET overall, how satisfied (very dissatisfied, somewhat dissatisfied, somewhat satisfied, or very satisfied) are you with your doctor's communication about your condition and its treatment?

Q43. Thinking about the doctor you see most often for your MF/PV/ET overall, how satisfied (very dissatisfied, somewhat dissatisfied, somewhat satisfied, or very satisfied) are you with your doctor's management and treatment of your MF/PV/ET?

Q44. How satisfied (not at all, only a little, somewhat, completely) are you with your MF/PV/ET doctor's understanding and support of your goals for treatment?

Q45. Slide the dot to represent who the main decision maker is when it comes to decisions regarding your MF/PV/ET treatment. (For example, if you feel it is evenly shared between you and your doctor then slide the dot to the middle.)

Q45a. Now slide the dot to best represent your preference for who the main decision maker should be when it comes to decisions regarding your MF/PV/ET treatment.

Q46. How did your doctor explain the potential MF/PV/ET symptoms you may experience and overall progression of the disease?

- They ran through a full and comprehensive list of symptoms
- They outlined symptoms I may experience
- They mentioned the top most bothersome symptoms I may experience
- They didn't discuss anything beyond symptoms I already have
- Other (specify)

Q46b. Please list those symptoms outlined by your doctor. <<allow 100 characters>>

Q46c. Please list those top most bothersome symptoms mentioned by your doctor. <<allow 100 characters>>

Q47. Based on your experience with the doctor you see most often for your MF/PV/ET, how much do you agree with the following statements on a scale from 1 (strongly disagree) to 4 (strongly agree):

Q47a. My doctor is genuinely concerned about helping me
Q47b. My doctor really listens to my concerns and addresses my questions
Q47c. My doctor keeps me informed about new treatment options
Q47d. My doctor involves me in decisions about my treatment
Q47e. My doctor has created a treatment plan or goals for therapy
Q47f. My doctor asks me about my symptoms at every appointment
Q47g. My doctor understands how much my condition impacts my life
Q47h. I don't feel comfortable discussing my symptoms with my doctor
Q47i. I feel I am burdening my doctor when I discuss how I am feeling
Q47j. I only discuss my symptoms when I am feeling really bad
Q47k. I only discuss my symptoms with the nurse
Q47l. There's not enough time during the appointment to discuss all my symptoms
Q47m. My doctor seems more interested in discussing blood counts
Q47n. I'm not certain how to describe my symptoms to my doctor

Q48. Within your current MF/PV/ET doctor's office, have you seen any other healthcare professionals aside from your MF/PV/ET doctor about your condition in the last 12 months?

Mark all that apply:

- Health care team (nurse practitioner or physician assistant)
- Clinic nurse
- Research nurse
- Nutritionist
- Psychologist
- Social worker
- Other (specify)
- Not seen any other healthcare professionals

Q49. Rank on a scale from 1 (not at all) to 5 (a great deal), the extent to which this healthcare professional is involved in counseling you regarding the treatment and management of your MF/PV/ET.

Q50. Did your doctor give you any information to learn more about your MF/PV/ET?

Q51. Did you, or your caregiver, do any research on your own to learn more about MF/PV/ET?

Q51a. Where did you find the most helpful information about MF/PV/ET?

Mark all that apply:

- Doctor's office
- Pharmacy
- Other patients
- Local patient support groups
- Online email discussions (advocacy listserv groups)
- Library
- Internet
- Other (specify)

Q51b. What sites do you go to most often for information about MF/PV/ET?

Mark all that apply:

- Health websites (WebMd, Health, Yahoo Health)
- Hospital websites (Mayo Clinic, Cleveland Clinic, etc.)
- Pharmaceutical company websites
- MPN advocacy group websites
- Other (specify)
- Don't know

Q52. How satisfied (very dissatisfied, somewhat dissatisfied, somewhat satisfied, or very satisfied) are you currently with how informed you are about MF/PV/ET?

Q53. How difficult (not difficult at all, slightly difficult, moderately difficult, or very difficult) was it to find good information about MF/PV/ET?

Q54. When was the most recent time you looked for information about MF/PV/ET or its treatment?

- Past week
- Past month
- Past 6 months
- Past year
- More than a year ago

Q55. Based on the results of your most recent search for information about MF/PV/ET, how much do you agree or disagree with each of the following statements on a scale from 1 (strongly disagree) to 4 (strongly agree):

Q55a. It took a lot of effort to get the information I needed
Q55b. I felt frustrated during my search for information
Q55c. I was concerned about the credibility of information
Q55d. The information I found was confusing and hard to understand
Q55e. I found a lot of good information that helped me better understand MF/PV/ET
Q55f. I prefer to learn about MF/PV/ET on the internet
Q55g. I prefer to receive educational materials in the mail
Q55h. I prefer to receive educational materials from my doctor

Q55i. I am more trusting of information given to me by my doctor

Q56. Do you have any sort of health insurance or health plan to cover your health care costs (yes or no)?

Q56a. What best describes your type of coverage?

- Group commercial insurance through employer or union
- Individual commercial insurance that's self-paid
- Medicare
- Medicaid or State assistance
- Tricare or VA benefit
- Other (specify)

Q56b. How much do you pay out of pocket monthly for prescription drug costs related to your disease?

- $0 to $25
- $26 to $50
- $51 to $75
- $76 to $100
- Over $100

Q58. Gender (male or female)

Q59. Are you of Hispanic, Latino or Spanish origin?

- No, not of Hispanic, Latino, or Spanish origin
- Yes, Mexican, Mexican American, Chicano
- Yes, Puerto Rican
- Yes, Cuban
- Yes, another Hispanic, Latino, or Spanish Origin

Q60. Select your race from the following (select one or more):

- White
- Black, African American, or Negro
- American Indian or Alaska Native
- Asian Indian
- Chinese
- Filipino
- Japanese
- Korean
- Vietnamese
- Other Asian
- Native Hawaiian
- Guamanian or Chamorro
- Samoan
- Other Pacific Islander

Q61. What is the last year or grade of school you completed?

- No high school
- Some high school
- High school graduate
- Technical post-secondary
- Some college
- Four year college graduate
- Post Graduate degree

Q62. What is your ZIP code?

Q63. Which of the following categories best describes your 2013 household income before taxes?

- $15,000 or less
- $15,001 to $25,000
- $25,001 to $35,000
- $35,001 to $50,000
- $50,001 to $75,000
- $75,001 to $100,000
- Over $100,000

Q64. Has anyone else in your family ever been diagnosed with MF, PV, or ET (yes, no, or don't know)?

Q65. Choose the statement which best describes how you completed this survey:

- I completed the survey myself.
- My caregiver completed the survey on my behalf.
